# The role of community participation for sustainable integrated neglected tropical diseases and water, sanitation and hygiene intervention programs: A pilot project in Tanzania

**DOI:** 10.1016/j.socscimed.2018.02.016

**Published:** 2018-04

**Authors:** Shirin Madon, Mwele Ntuli Malecela, Kijakazi Mashoto, Rose Donohue, Godfrey Mubyazi, Edwin Michael

**Affiliations:** a Department of International Development & Department of Management, London School of Economics & Political Science, UK; b National Institute for Medical Research, 2448 Ocean Road, P.O. Box 9653, Dar es Salaam, Tanzania; c Department of Biological Sciences, University of Notre Dame, Notre Dame, IN 46556-0369, USA

**Keywords:** NTD-WASH programs, Health program sustainability, Community participation, Village governance, Mixed methodology, Tanzania

## Abstract

Strategies aimed at reducing the prevalence of neglected tropical diseases (NTDs) in Tanzania including those attributed to water, sanitation and hygiene (WASH) problems have been largely top-down in nature. They have focused on strengthening the governance of NTD-WASH programs by integrating different vertical disease programs and improving the efficiency of report-generation. In this paper, we argue for community participation as an effective strategy for developing sustainable village health governance. We present the results of a pilot undertaken between November 2015 and April 2016 in which we adopted a mixed methods case study approach to implement an Enhanced Development Governance (EDG) model using existing village governance structures. Our results show that the EDG model was associated with a statistically significant reduction in the prevalence of schistosomiasis and diarrhoea, and has led to an increase in awareness of WASH interventions for sustaining gains in NTD control. We identify five key social processes enacted by the EDG model that have led to improved health benefits related to frequency of meetings and attendance, promotion of health and sanitation awareness, income-generating activities, self-organising capabilities, and interaction between village bodies. These findings hold important implications for conceptualising the role of community participation in sustaining NTD-WASH intervention programs and for sensitising institutional and policy reform.

## Highlights

• Community participation plays an important role in sustaining improved health outcome.
• We identify five key social processes that were enacted during program implementation.
• The EDG model is associated with a statistically significant but small decrease in schistosomiasis and diarrhoea.
• The study provides an innovative way to financially support community health workers.

## 1 Introduction

In Tanzania, the prevalence of neglected tropical diseases (NTDs) remains of major concern with over 40 million people in 2013 requiring mass drug administration (MDA) for onchocerciasis, lymphatic filariasis, soil-transmitted helminths, schistosomiasis and trachoma (Mwingira et al., 2016). While MDA is the primary strategy for controlling schistosomiasis and other NTDs, by not addressing the fundamental determinants of NTDs, continued re-exposure allows rapid reinfection implying the need for a high level of coverage for many years to achieve successful control and elimination. However, sustaining high coverage of the target population is challenging for control programs due to resource limitations, local resistance, non-compliance, and waning community and political support resulting in growing awareness of the need to improve the governance and delivery of NTD programs (Bardosh, 2014). In 2005, the introduction of an integrated NTD control program in Tanzania provided an opportunity to minimise costs, streamline program activities and maximise the use of resources (Kabataraine et al., 2010). Efforts have also been made to define the role played by Community Health Workers (CHWs) in Tanzania and other Sub-Saharan African countries as these voluntary agents deliver relatively similar interventions across several programs to the same people (Marchal et al., 2011). From the mid-1990s, the introduction of a health management information system (HMIS) and more recently a mobile-phone based NTD HMIS piloted in Mkuranga district of Tanzania led to improvements in data-handling, although these systems have not resulted in increased local voice and participation in health planning and decision-making (Madon et al., 2014).

The NTD program governance strategies described above required considerable power to be vested with district-level agencies for strengthening the coordination of different programs, resources and information. A more recent strategy adopted by the Tanzanian government, the focus of this paper, has been to concentrate on improving governance and accountability of NTD control and WASH programs at the village level. There are several drivers for pursuing such an approach. First, NTDs are influenced by water, sanitation and hygiene conditions requiring joint implementation, management and evaluation of NTDs and WASH programs as part of an overall village development agenda (APPMG, 2015). Second, while the district holds substantial decision-making power with regards to NTD and WASH programs execution and coordination, the effectiveness of both programs has been impaired as their actual implementation occurs at the village level and requires active community participation (Maluka and Bukagile, 2016). Third, while recognising the important role played by CHWs in providing healthcare to the local community, the government needs to find locally-generated ways of providing income to these frontline workers. Non-financial incentives, such as improved working conditions or training and career path incentives, have been put in place in east and southern African countries (Dambisya, 2007) while in other cases local financial solutions have been sought; in India, for example, CHWs are paid through a fee-for-service system (Singh, 2013). The Tanzanian government's current village-level focus on NTD-WASH programs draws inspiration from the Mtwara Model launched in 2002 by the MoHSW^1^ aimed at improving local health governance in the Mtwara region which was endemic for lymphatic filariasis. The model was implemented as a local government initiative with the Mtwara Regional Commissioner supporting each district in the region to allocate a budget to supplement central government funds for activities aimed at eliminating lymphatic filariasis. The Commissioner paid regular visits to each district to inspect the implementation of the program and was actively involved in raising awareness amongst villagers about the necessity of eliminating lymphatic filariasis. As a result of this local initiative, MDA coverage improved and the prevalence of lymphatic filariasis declined significantly over the years in that region (Malecela et al., 2013).

This paper evaluates the recent experience of resurrecting village governance structures in Tanzania based on an exploratory study undertaken between November 2015 and April 2016 in four villages in Rufiji District as part of a pilot project launched by the MoHSW. The aim was to create a platform for a broad, interactive discussion on NTDs and WASH programs and their joint implementation, management and evaluation at village level. The project involved the development of a model for sustainable village governance referred to as the Enhanced Development Governance (EDG) model to address the deficiencies in the existing NTD control program. The EDG model which was based on existing village structures such as the Social Services Committee (SSC) and village community bank (VICOBA)^2^ was provided with resources to promote community awareness of NTD prevention and control by establishing new mechanisms for participation in village health.

There are two key objectives in this paper, namely (1) to describe the new mechanisms for community participation that emerged as a result of the EDG model, and (2) to test the hypothesis that the EDG model effectively improved health outcomes and program sustainability. In the next section, we draw from literature on community participation for health program sustainability as a relevant theoretical lens for studying the EDG intervention in Tanzania. Following a description of our methodological approach, we present the results from our evaluation exercise focusing on two key aspects. First, we demonstrate the extent to which the EDG model has increased the opportunities for local people to raise their voice and participate effectively in health planning. Second, we identify whether the enactment of this new structure has resulted in the reduction of the prevalence of one selected NTD (schistosomiasis) as well as diarrhoea in our study villages and more broadly in improvements in health-related outcomes. Finally, we draw on our study findings to discuss theoretical and policy-related implications associated with supporting sustainable village governance structures for NTD-WASH control in a low-income country like Tanzania.

## 2 Conceptualising community participation in health program sustainability

The issue of health program sustainability has received attention over the past few decades as an important topic in both academic and policy literature. The concept is broadly accepted as referring to the continued use of program components and activities for the achievement of desirable or intended health outcomes beyond the initial funding period (Shediac-Rizkallah and Bone, 1998; Scheirer and Dearing, 2011). Within this discourse, the issue of what exactly is to be sustained has been a recurrent theme. From a medical or health systems perspective, of importance has been to track the long-term health effects of the program and for its institutionalisation within the pre-existing structures and processes of the health system (Pluye et al., 2004; Shigayeva and Coker, 2015). In their study of a community-based dengue control intervention in three health zones in Santiago de Cuba (Cuba), Toledo Romani et al. (2007) analysed the maintenance of health benefits and the level of institutionalisation of the intervention within the health system, for example in terms of earmarking funding on an ongoing basis for the execution of the new program.

Since Alma Ata,^3^ a growing strand of literature sees community participation as an essential driving force for health program sustainability based on the assumption that working with communities can help make interventions more relevant to local priorities (Rifkin, 1986, 2014; WHO, 2002; Draper et al., 2010). However, there remains lack of conceptual clarity about how exactly community participation leads to sustainable health outcomes (Hossain et al., 2004). An important debate in the literature has revolved around whether the aim of community participation should be to improve the efficiency of service delivery by increasing the uptake of interventions, or whether it should be linked to addressing broader structural issues of equity in healthcare (Rifkin, 2003; George et al., 2015a). Much effort has been dedicated towards understanding community participation as a process from low levels of participation to higher levels influenced by the socio-economic and political context within which it is embedded, as illustrated, for example, by Frumence et al. (2014) in their study of participation in health facility governing committees in Tanzania. The spidergram model developed by Rifkin et al. (1988) has been widely used to conceptualise community participation as a process influenced by different factors such as needs, leadership, program organisation, management and resource mobilisation, which taken together measure the uptake and sustainability of the health intervention. The model visualises each indicator separately as a continuum from narrow to wide participation, which is then linked to the rest of the indicators to arrive at an overall assessment of how community participation influences program sustainability.

There is increasing recognition in the literature that community participation by its very nature needs to address issues of power and control. For example, lessons from a study of community-directed treatment programs for onchocerciasis control across four African countries revealed the need for program implementers to improve communication strategies so that communities have greater control to plan the timing of treatment interventions and to take ownership of the intervention (Amaziqo et al., 2002). Health program sustainability may also be compromised due to lack of understanding or trust between the community and frontline health workers as well as due to the capacity and motivation of community health workers. In a study undertaken in Uganda, unrealistic community expectations, limited drugs and supplies, poor supervision and lack of compensation resulted in feelings of disempowerment amongst health workers, which ultimately adversely affected their motivation to deliver services for febrile children (Banek et al., 2015). Issues of power and control have led to an extension of the initial spidergram model drawing on social psychology theory to study how social norms and behaviour affect community participation and program sustainability (Campbell, 2013).

While there has been increasing interest in understanding how community participation influences health program sustainability, most studies have been undertaken at a point in time rather than capturing changes over time (Draper et al., 2010). Specifically, to date there has been no study of community participation in health program sustainability with respect to NTD control in the context of a developing county - a gap we attempt to address in this paper. We study a pilot project introduced in Tanzania in 2015 called the EDG model which used existing village governance structures to improve NTD-WASH control through community engagement. Although a pilot project with a finite end date, the enactment of social processes during the implementation phase of the project holds important implications for program sustainability (Pluye et al., 2004). While village health committees were formally constituted in Tanzania in 1982, there was low recognition amongst community members of their existence, mandate and functioning (Mubyazi et al., 2007). More recently, there has been political will to resurrect these structures through training and capacity-building (Maluka and Bukagile, 2016).

## 3 Methods

### 3.1 EDG model and intervention

#### 3.1.1 Existing village government structure

In both districts, the village council is mandated to meet monthly and comprises of approximately 25 members, including hamlet and village council chairpersons who are elected during local government elections held every five years, and the village executive officer VEO. The village council has three committees of 8 members each: the SSC, the finance and planning committee, and the safety committee. The SSC is charged with the responsibility of land, education, water, health and environment issues with committee members who are nominated based on age, experience, gender, discipline, trustfulness in the community and literacy endorsed at community-wide quarterly village general assembly meetings.

The VEO is the secretary of the SSC and is responsible for taking minutes, maintaining records and feeding the village council with the SSC report for decision making and approval. However, in both districts, lack of funds and leadership induction training to SSC members has contributed to lack of understanding of their roles and responsibilities, and ultimately poor performance of these committees.

#### 3.1.2 Description of the EDG model

**Fig. 1:**
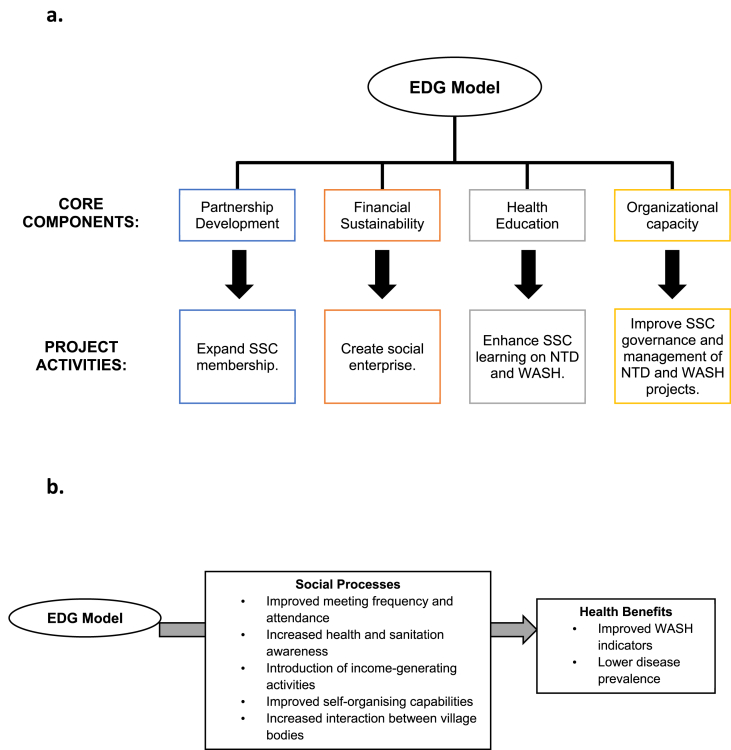
Overview of the Enhanced Development Governance (EDG) model: (a) Core components and project activities of the EDG model; (b) Conceptualising the linkage between the EDG model, social processes, and NTD Control.

The EDG model comprised four core components: partnership development, financial sustainability, health education, and organizational capacity, with a list of key activities for implementing each component (Fig. 1a).

To enhance partnership development, the EDG model sought to expand membership of the SSCs by co-opting additional relevant villagers through a process that consisted of several activities. These included holding initial meetings with village council members in the four intervention villages to explain the objectives and timeframe of the pilot. The village council members were requested in advance by the project team to act as core members of the pre-existing SSCs and to help identify additional relevant members. Several measures were undertaken to ensure that SSC members were inclusively selected. Villages normally have two CHWs and both were co-opted as SSC members. In villages with more than one VICOBA, a representative member from each group was selected and the same procedure was employed for youth and religious groups. School teachers responsible for WASH and health were nominated by the head teacher and Community Drug Distributors were randomly selected by a village council member from a list of all distributors in a particular village. These individuals identified as key informants in the study were then co-opted as new SSC members. Table 1 compares the composition of the village committees pre and post intervention.

**Table 1:** Composition of the SSCs in the intervention wards before and during study implementation.

	Pre	Post
Social Services Committee members	8	8
Village government staff (VEO and chairperson)	0	2
VICOBA groups representatives	0	5
Religious leaders	0	5
NGO/Civil society representatives	0	1
CHWs	0	4
Teachers	0	1
Youth representatives	0	1
Politicians	0	1
Total	8^a^	28

a Same number of SSC members for non-intervention villages.

To ensure financial sustainability of the strengthened SSCs, the project team provided start-up funds and training for SSCs to conduct income-generating activities. Additionally, funds were provided for NTD-WASH activities held in the intervention districts based on the overall project grant fund of USD 100,000. The budget was discussed with each village council during the introductory phase of the project and SSCs were asked to provide a budget estimate for NTD-WASH and income-generating activities. Based on their response, a sum of USD 19,511 (which included a start-up fund of USD 1603) was collectively agreed upon for intervention activities in each village.

Health education was introduced as a core component of the model to educate the SSC on disease prevention and WASH in an effort to disseminate this knowledge to the communities. Initially, the project team provided NTD-WASH education to the committee members through lectures and discussion and educated the SSCs on the implementation and monitoring of NTD and WASH projects as well as on budgeting.

### 3.2 Study design and analysis

In this paper, we followed a mixed methodology study design which has become increasingly popular in health systems research in which qualitative approaches are used to explain quantitative results and to help develop effective interventions (Ozawa and Pongpirul, 2013).

#### 3.2.1 Study Design

We used a case control design to conduct this study. The study was carried out from November 2015 to April 2016 in two districts, namely Rufiji (as intervention district) and Mkuranga (used as a control) in the Coast (Pwani) Region of Tanzania, which demonstrate comparable demographic, socioeconomic, political and WASH indicators as shown in Table 2. These districts are located along the coast of the Indian Ocean in Tanzania and both have a high prevalence of NTDs such as schistosomiasis, trachoma, lymphatic filariasis and soil-transmitted helminths (SCI/MoHSW, 2010). The majority of the people in Rufiji and Mkuranga are Moslems with few Christians and followers of traditional religions. In both districts the majority of the people are subsistence farmers. Tap water supply is very limited and the majority of people rely on communal boreholes or natural spring water for domestic purposes, while a few use harvested rainwater. Waterborne diseases such as cholera and diarrhoea are the major health problems in the area as reported through the health services and as perceived by local people.

**Table 2:**
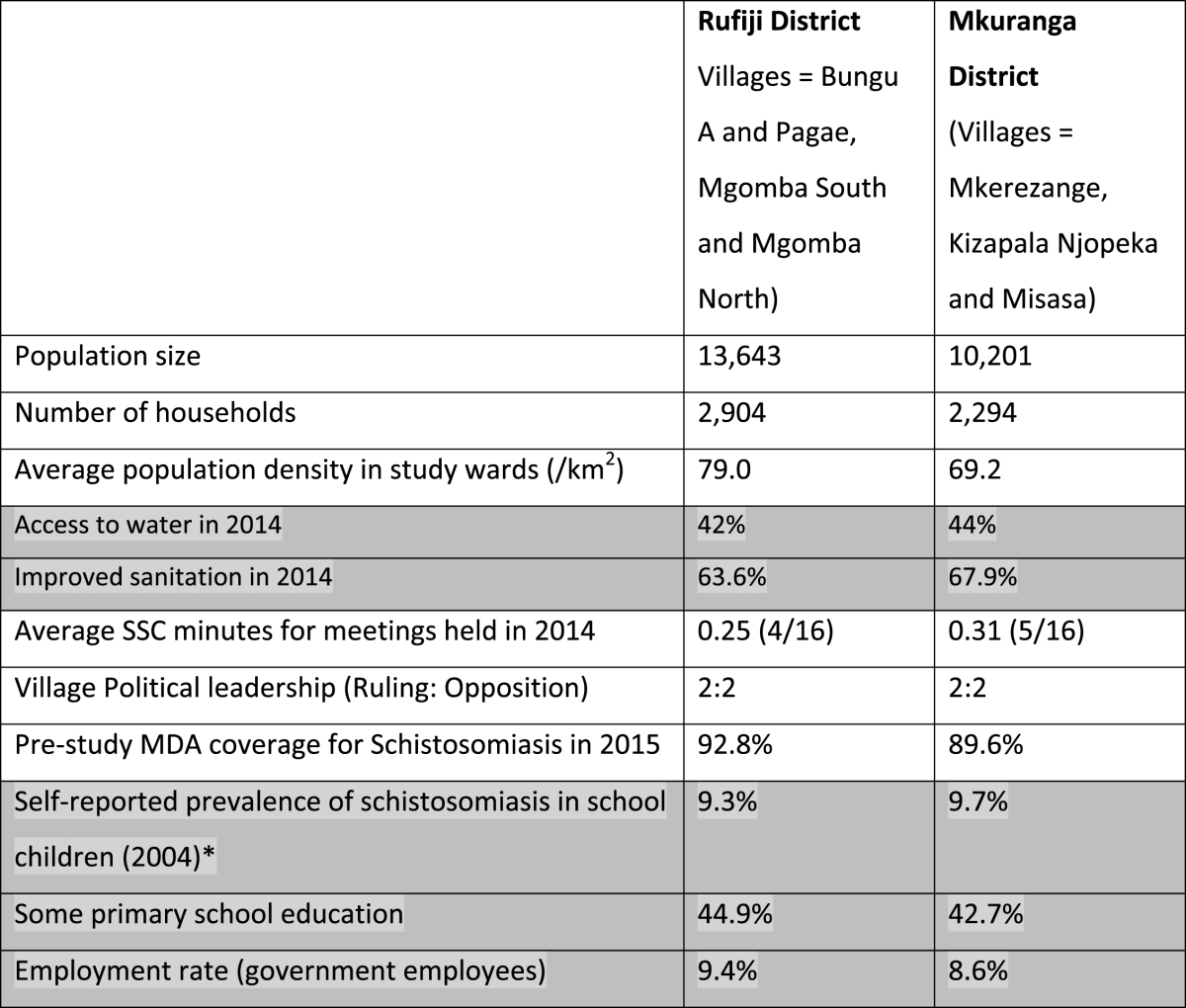
Key Contextual Information for Rufiji and Mkuranga districts.

Shaded categories show district-level information.

Control villages were purposefully not selected from the same district to avoid spill-over of the intervention to the control villages as members of the village bodies were also members of ward development committees.

#### 3.2.2 Sampling approach

The villages for the study were identified through a multistage simple random sampling technique whereby in each district we identified two wards and then randomly selected two villages in these ward. For the intervention district, the four selected villages were Mgomba South, Mgomba North, Pagae and Bungu. The control villages included Niopeka, Misasa, Mkerezange and Kizapala.

The individuals identified as key informants for the study were purposefully selected based on their roles at the ward, village and NGO levels. For the quantitative household and school-based surveys, sample sizes were calculated based on previous prevalence estimates of diarrhoeal diseases and schistosomiasis, respectively, using a 95% confidence interval, 2% acceptable margin of error, and a 10% non-response rate (Mashoto et al., 2014). For the household surveys, VEOs in collaboration with hamlet chairpersons produced lists of all households with at least one child under-5 in their respective villages, from which simple random sampling procedure was employed to select households. For the school surveys, random sampling procedure was also used to select pupils from class attendance registers. A proportion to population sample size was collected for the survey employed to assess community knowledge on WASH and NTDs in intervention villages, from which respondents were selected using simple random sampling.

#### 3.2.3 Qualitative data collection and analysis

Using participatory rural appraisal (PRA) techniques including resource and income mapping along with in-depth interviews (IDIs) and focus group discussions (FGDs), committee members’ perspectives were elicited on the adequacy of existing NTD and WASH delivery mechanisms and on the new social processes that were being enacted through the EDG model (Fig. 1b). The project team attended monthly committee meetings as participant observers but also intentionally provided immediate feedback on how each committee was performing in terms of attendance rate, agenda preparation, minute-taking and follow-up on issues discussed at the previous meeting, budgeting and planning. Through FGDs, members of the enhanced SSCs brainstormed on the opportunities and challenges of using resources optimally for NTD-WASH activities in their village. The SSCs were subsequently supported by the project team to organise NTD prevention and WASH awareness campaigns.

Our interviews and FGDs enabled comparison of the different aspects of NTD-WASH governance across the villages according to a thematic coding scheme. We formulated initial codes from the transcripts as closely as possible to the broad themes identified from the literature review, namely community organisation, leadership, resource allocation, power struggles and social norms. Subsequently, during the conduct of our fieldwork as interesting stories emerged we began to cluster these initial codes and identify new sub-codes which eventually provided a structure for presenting our qualitative results. To counter against possible bias in the choice of codes, all the authors were involved in this coding exercise.

#### 3.2.4 Quantitative data collection and analysis

Household and school-based surveys were conducted to collect quantitative data on health-related outcomes in both districts before and after implementation of the EDG model (but data on NTD prevention and WASH knowledge was collected only from the intervention villages where this education was provided). Baseline data was collected in February 2015 while post-intervention data was collected in April 2016.

The household survey assessed the 1-week prevalence of diarrhoeal diseases among children under-five through interviewer-facilitated structured questionnaires administered to the head of household. Diarrhoeal disease is defined in this study using the standard WHO definition of having 3 or more loose stools in a 24-hour period. Additionally, interviewers inspected and collected information on household sanitation and hygiene facilities. The school survey used self-administered questionnaires, based on the review conducted by Lengeler et al. (2002), to assess health-related outcomes including two forms of schistosomiasis: urinary caused by *S. haematobium* and intestinal caused by *S. mansoni*. A full description of the methodology for the classification of schistosomiasis is provided in Appendix A.

All quantitative analysis was conducted using R version 3.1.1 (R Core Team, 2014). To evaluate the effect of the intervention on dichotomous outcome variables over time, we utilized logistic regression models with an interaction term between time and intervention. Given the clustered nature of our data, with individuals grouped into schools or villages from which repeated cross-sectional surveys were conducted, the generalized estimating equation (GEE) methodology was used as described in the Appendix B (Liang and Zeger, 1986). An exchangeable correlation structure was specified for each model. As a small number of clusters (≤30) can lead to biased variance estimates using the robust sandwich variance estimator, we used an alternative jackknife variance estimator. Due to baseline factors such as sex, age, and socio-economic status not being found to differ significantly between the control and intervention districts, the models were not adjusted for such confounders (see Appendix C). All GEE analyses were conducted using the “geepack” package in the R programming language (Hojsgaard et al., 2006).

#### 3.2.5 Research ethics

Ethical clearance was sought and permission obtained from the National Health Research Review Committee. Respondents were explained the scope of the study in local language and every effort was taken to respect the customs and culture of the people of both intervention and control districts.

## 4 Results

### 4.1 Qualitative results

Our qualitative inquiry focused on the process of SSC formation and implementation over the six-month duration of the project.

### 4.2 Meeting attendance and frequency

The quorum of members who attended SSC meetings included the SSC Chairperson, Village Council Chairperson, VEO, Ward Executive Officer (WEO), VHWs, religious leader(s), primary school teacher, a VICOBA member, hamlet chairperson, SSC Secretary and SSC members. Over time, the attendance rates seem to have stabilised around 15–25 core and co-opted members as shown in Table 3.

**Table 3:** SSC Membership and Meeting attendance (Nov 2015–April 2016).

Village name	Membership		SSC members meeting attendance per month					Meetings per month
	Proposed	Actual	1^st^ Month	2^nd^ Month	3^rd^ Month	4^th^ Month	5^th^ Month	Average number
Mgomba South	28	26	25	25	26	20	25	2
Mgomba North	25	19	19	19	16	18	15	2
Bungu	26	21	18	21	11	15	15	1.5
Pagae	27	21	19	21	16	17	18	1.5

Despite the SSCs being mandated to meet once every month, Table 3 illustrates that in all four study villages committee members met more regularly to discuss the initiation of income-generating activities that were designed to provide income to health workers who were so far unpaid and for helping to finance NTD-WASH activities. In total, twenty-four SSC meetings were held in the intervention district together with display of posters in the four village offices and circulation of four hundred leaflets to villagers in the district.

### 4.3 Promoting health and sanitation in the community

Activities undertaken by the SSCs to promote health and sanitation in the community included providing health awareness education sessions, organising village clean-up operations and enforcing penalties to households for non-complicance with the sanitation and hygiene standards set by the SSC as discussed below:

#### 4.3.1 Conducting health and sanitation awareness education

While education awareness sessions were held at the beginning of the pilot, it was only from the third month that regular sessions were held in individual households and public spaces such as hamlets, health facilities and schools. By the fourth month of the pilot project, SSC members in Mgomba South and Mgomba North scheduled two sessions on particular days of the month while one such session was organised in Pagae and Bungu.

In all villages, there was a strong community perception that the regularity of imparting health and sanitation awareness education with trainers working alongside SSC members was helping to educate the community and reduce power struggles between the implementers of reform and the community. This came across in a statement made by the VEO of Mgomba South after implementation of the EDG model,

‘There is a noticeable difference – nowadays people are being sensitized because they have received education and seem to understand the importance of taking these medicines [referring to MDA drugs]. So after education and sensitisation sessions the educators walked from one street to another together with our committee’.

#### 4.3.2 Organising village clean-up operations

By the fifth month of the project, village clean-up operations were routinely organised by the committee members backed by the village council on a specific day of the month in public places. The exercise involved cleaning water wells and roads as well as areas surrounding households, schools, health facilities, village offices and markets. Individual SSC members were assigned responsibility to oversee and participate in the clean-up operation within their areas of jurisdiction. By the fifth meeting in Pagae, hamlets started to organise the clean-up activity on a weekly basis. Community members were generally positive about recognising these activities as a shared responsibility of both the committee and villagers. Evidence of improved trust relations between the SSC and community members is demonstrated in the following remark from a FGD in Mgomba North which was also strongly endorsed by discussants from other villages:

‘Implementers of improved health and sanitation are both the community and the SSC. The role of the SSC has been to sensitise the community to keep their plots clean. The SSC also engages in implementation together with the community’.

#### 4.3.3 Enforcing compliance to sanitation and hygiene standards

By the fourth month, all four villages had completed a survey to identify households with no/poor latrine to whom penalties were enforced to ensure standards were observed as in Mgomba South where three members of the community were fined Tanzanian shillings 10,000/- (USD 4.6) for failing to clean the area surrounding their households on time. However, in the other villages, SSC members were concerned about administering fines to people who might have genuinely made some efforts to clean up but may not have met the standards specified. Below is a remark made by the Mgomba North Chairperson who felt that the real mandate of the SSC was to educate villagers rather than punishing them,

‘The main cause for the change in attitudes towards improved sanitation is not that they are scared of the law but that once people saw the cholera outbreak occurring they started constructing their own toilets’.

### 4.4 Introducing income-generating activities

The enhanced SSCs assumed responsibility for introducing and sustaining income-generating activities to support health workers and to help finance NTD-WASH activities in the village. By the end of six-months, viable income-generating activities such as selling of soap, sugar, vitenge (cloth), chicken-rearing and money lending, had become institutionalised in most intervention villages. While it was difficult to obtain accurate data on the revenue generated from these activities, it was clear that income-generating activities were becoming a mainstay of the SSC committees as reflected in the following statement made during a FGD in Bungu village,

‘Those people who came from the ministry funded us and told us to choose projects and in response we chose two projects, to make soaps and produce poultry of the local breed type. We now depend on the continuation of these projects so even if NIMR leaves us we will continue. Ever since the day they came and we sat together, I believed that they are just facilitating us to help us know our responsibilities’.

Income-generating activities have helped to motivate and empower CHWs with resources as indicated by the following quotes from the Village Council Chairpersons in Bungu and Mgomba South,

‘You see, other things which motivates them (CHWs) is when they were told that they will also receive start-up fund to initiate income-generating activities and that the profits will be used to support implementation of SSC activities and as incentives for committee members’. (Bungu)

‘The strategy used by the project team required us to come up with our own business idea and we are thankful because we managed to do well and this really helped us a lot. It became like a motivation for SSC members to increase their enthusiasm in carrying out their responsibilities and roles effectively. So many activities within a short time and SSC became live’. (Mgomba South)

### 4.5 Strengthening self-organising capabilities within the SSC

#### 4.5.1 Meeting execution

By the fifth month of the project, agenda-setting, minute-taking and follow-up action had improved. There was also a heightened sense of obligation for members to attend meetings regularly, for example non-attending SSC members from Pagae were replaced by the hamlet chairperson.

#### 4.5.2 Data collection

Data collection remained sporadic in the majority of SSCs. By the fifth month of study, only Mgomba South could demonstrate the ability to report on the status of sanitation and hygiene in the village, the amount of coverage achieved during NTD MDA exercises, household compliance with hygiene and sanitation standards, and NTD-WASH education in the village.

#### 4.5.3 Transparency and accountability

The lack of transparency and accountability in income and expenditure affected trust relations among SSC members. During a village meeting held in Mgomba South in March 2016, we observed how lack of disclosure by VEO and hamlet chairperson about the receipt of fines imposed on the community for poor sanitation led to suspicion that that money was being usurped by powerful members in the village. In contrast, although poultry-rearing income generation activity had been established within the SSC Secretary's personal compound in Bungu, a FGD conducted in the village in April 2016 made clear that the village council had suggested that a contract be drawn up to increase transparency,

‘In our SSC meeting, there are things we forward to the village council and they give their opinion. For example, last time they told us we are constructing a house but we don't have a contract with the land owner so we should have a written contract so that it shouldn't look like everything is for the owner’.

### 4.6 Interaction between village bodies

From the third SSC meeting, all four villages communicated with the village council for financial support towards the cost of cleaning the school site, clearing garbage, constructing wells and building latrines. Although funds were unavailable, the EDG intervention has helped to raise local government awareness about the importance of having a budget and setting aside funds for such items. In other cases, the SSC called the village or ward council to seek advice on encouraging villagers to help with clean-up programs, asking well-owners to provide services to villagers, and addressing issues that cross-cut several villages such as sanitation or crime. As members in a FGD at Bungu village emphasised,

‘We sit as a committee and discuss issues but if we need special advice, we take it to the village council’.

More regular interactions between the SSC and the village general assembly, health facilities, livestock and water experts also suggest a greater sense of empowerment as the SSC is perceived as a credible village body.

### 4.7 Quantitative results

#### 4.7.1 Effect of the intervention on disease prevalence

As illustrated in Table 4, the intervention was associated with a significantly larger reduction in the questionnaire-based prevalence of *S. mansoni, S. haematobium,* and parasitic infection when compared to the control district.

**Table 4:** Comparison of the questionnaire-based prevalence of schistosomiasis among schoolchildren between intervention and control districts at baseline and follow-up.

Outcome indicator	Intervention		OR^a^	Control		OR^b^	ROR^c^ (95% CI) ^d^	Intervention effect
	Pre	Post		Pre	Post			
	n = 894	n = 945		n = 810	n = 833			
	%	%		%	%			
Schistosomiasis	11.4	7.0	0.55*	8.1	7.0	0.83	0.67 (0.48–0.93)*	Significantly larger ↓
*S. mansoni*	2.7	1.6	0.56*	1.6	2.4	1.49	0.37 (0.22–0.64)*	Significantly larger ↓
S. *haematobium*	10.5	6.3	0.55*	7.0	6.2	0.87*	0.63 (0.44–0.91)*	Significantly larger ↓

^a^ Odds ratio (OR) for the change in outcome from baseline to follow-up in the intervention group only. Obtained from logistic GEE model. Shaded boxes indicate statistical significance at p < 0.05.

^b^ OR for the change in outcome from baseline to follow-up in the control group only. Obtained from logistic GEE model.

^c^ Ratio of Odds Ratios (OR^a^/OR^b^). Obtained from logistic GEE model.

^d^ 95% Confidence Interval (CI) obtained using jackknife variance estimation.

*Statistical significance at p < 0.05.

Table 5 illustrates that both the control and intervention districts demonstrated a similar significant reduction in the one-week prevalence of diarrhoeal-disease, so the intervention did not have a significant effect overall. As the reasons behind this situation were not obvious, two possible explanations have to be considered. First, a separate intervention, the National Sanitation and Hygiene Campaign which started in December 2015 exclusively in the control district may have caused the reduction in diarrhoea prevalence in the control district.^4^ Second, a cholera outbreak in Rufiji and Mkuranga districts which started in February 2016 may have led to intensified WASH campaign and use of chlorine-based water treatment products and confounded the intervention effect.

**Table 5:** Change in 1-week diarrhoeal prevalence outcome in intervention versus control districts at baseline and follow-up.

Outcome indicator	Intervention		OR	Control		OR	ROR (95% CI)	Intervention effect
	Pre	Post		Pre	Post			
	n = 934	n = 870		n = 770	n = 844			
	%	%		%	%			
Diarrhoea prevalence in children under-5	10.3	4.7	0.44*	11.8	4.3	0.35*	1.29 (0.57–2.87)	–

*Statistical significance at p < 0.05.

#### 4.7.2 Effect of the intervention on imparting community knowledge about NTD control

Comparing the period before and after the EDG model intervention, Table 6 shows that community members in the intervention district demonstrated significant improvements in knowledge on NTD-WASH aspects We used more than one item to assess knowledge on different topic areas^5^ as described in more detail in Appendix D.

**Table 6:** Proportion of community participants in the intervention district correctly answering each question item in NTD-WASH topic areas before and after the intervention.

Knowledge on:	Before Intervention (n = 1451)	After Intervention (n = 1700)	χ^2^ value	p-value
	Number (%)	Number (%)		
Causes of water contamination	1075 (74.1)	1418 (83.4)	40.6	<0.001*
Water for NTDs prevention	21 (1.4)	343 (20.2)	266.9	<0.001*
Hygiene and sanitation for NTDs prevention	829 (57.1)	1295 (76.2)	128.3	<0.001*
Commonly occurring NTDs	220 (15.2)	459 (27.0)	64.2	<0.001*
NTDs that lead to disability	48 (3.3)	613 (36.0)	504.5	<0.001*
NTDs transmission	17 (1.2)	701 (41.2)	711.9	<0.001*
WASH importance for NTDs prevention	13 (0.9)	190 (11.2)	135.6	<0.001*
Overall	20 (1.4)	481 (28.3)	422.1	<0.001*

*Statistical significance at p < 0.05.

Table 7 shows that intervention villages experienced a significantly larger increase in the proportion of students identifying disease prevention as a reason for hand washing when compared to control villages [ROR = 5.78 (2.33–14.33)]. Additionally, compared with their counterparts in control villages, heads of households in the intervention villages were significantly more likely to identify germ-killing as a reason for handwashing at follow-up. There was a significantly larger increase in the intervention district compared to the control district [ROR = 7.16 (2.96–17.31)].

**Table 7:** Change in indicators in intervention versus control districts at baseline and follow-up.^a^

Outcome Indicator	Intervention		OR	Control		OR	Ratio of Odds Ratios (95% CI)	Intervention effect
	Pre	Post		Pre	Post			
	*n*=*934*	*n*=*870*		*n*=*770*	*n*=*844*			
	%	%		%	%			
b. Effect of the intervention to impart community knowledge on NTD control								
Reasons for HW: to prevent disease^+^	74.4	89.7	2.96*	78.2	66.1	0.51	5.78 (2.33–14.33)*	Significantly larger ↑
Reasons for HW: to kill germs	43.7	74.8	3.85*	77.0	66.7	0.54	7.16 (2.96–17.31)*	Significantly larger ↑
c. Effect of the intervention on sanitation and hygiene behaviours								
Family defecation location: latrine^+^	97.8	98.6	1.66	98.9	97.4	0.41*	4.01 (1.23–13.07)*	Significantly larger ↑
Faeces present around compound	7.8	6.3	0.84	11.0	0.7	0.06*	13.84 (1.53–125.12)*	Significantly smaller ↓
Wash hands with running water	22.8	51.7	3.60*	42.7	51.1	1.56	2.30 (1.00–5.32)*	Significantly larger ↑
Handwashing at key events								
After using latrine	88.3	94.0	2.20*	94.0	95.0	1.18	1.87 (0.91–3.84)	–
After cleaning child's bottom	20.6	52.1	4.09*	63.6	52.5	0.59	6.88 (2.13–22.29)*	Significantly larger ↑
Before preparing food	18.6	37.6	2.57*	50.6	50.4	0.94	2.72 (0.76–9.79)	–
After preparing food	17.9	35.6	2.47*	46.1	50.2	1.08	2.29 (0.74–7.06)	–
After handling dirty things	32.2	44.4	1.71*	70.0	52.3	0.44*	3.92 (2.31–6.65)*	Significantly larger ↑
Rarely/never use soap during HW	33.1	20.2	0.53*	22.3	23.7	1.07	0.49 (0.27–0.92)*	Significantly larger ↓
d. Effect of the intervention on household ownership and quality of WASH-related assets								
Ownership of utensils rack	19.3	61.7	6.08*	36.2	51.2	1.86*	3.27 (1.81–5.88)*	Significantly larger ↑
HW facility outside latrine	4.0	21.7	6.53*	39.2	31.3	0.68	9.66 (1.55–60.15)*	Significantly larger ↑
Drop hole covered	38.0	47.7	1.44	56.8	38.0	0.49*	2.93 (1.27–6.88)*	Significantly larger ↑
Good latrine privacy	2.3	9.9	4.62*	10.5	10.9	1.24	3.73 (1.64–8.53)	Significantly larger ↑
Standing wall	10.8	47.8	7.13*	19.0	35.5	2.92*	2.44 (0.95–6.29)	–
Lockable door	7.0	33.1	6.37*	22.7	16.1	0.73*	8.74 (6.18–12.36)*	Significantly larger ↑
Roof	2.4	19.9	9.55*	20.4	23.8	1.42*	6.71 (4.50–9.99)*	Significantly larger ↑

^a^We are not aware of any other sanitation campaign being implemented in the control or intervention districts during the period of time post-baseline data collection and pre-intervention. The National Sanitation and Hygiene Campaign referenced in the paper began in December 2015, so this would not have affected any of the baseline indicators pre-intervention. Therefore, we do not have any reason to believe any major changes in sanitation and hygiene occurred between February and November 2015. With regards to disease outcome, both schistosomiasis and diarrhoeal diseases are chronic endemic infections and therefore we would not expect any significant changes to occur between the two dates.

^+^ Question from school-based survey; sample size pre/post intervention is 894/945; sample size pre/post control is 810/833.

*Indicates statistical significance at p < 0.05.

#### 4.7.3 Effect of the intervention on sanitation and hygiene behaviour

Table 7 shows that the intervention was associated with a significantly larger increase in the odds of pupils’ families using a latrine for defecation [ROR 4.01 (1.23–13.07)]. Both districts saw decreases in the presence of faeces around the compound (a marker for open defecation); however, this decrease was larger in the control district [ROR = 13.84 (1.53–125.12)].

Handwashing practices seemed to improve more in the intervention villages than control villages as demonstrated by the following indicators: use of running water instead of communal pots, handwashing frequency at key events, and use of soap. A significantly larger increase in handwashing with running water as opposed to using a communal pot was noted in the intervention villages when compared to the control district [ROR = 2.30 (1.00–5.32)]. The odds of handwashing were found to be significantly higher in the intervention villages than in the control ones at key events including the following: (a) after cleaning a child's bottom [ROR = 6.88 (2.13–22.29)], and (b) after handling dirty things [ROR = 3.92 (2.31–6.65)]. Finally, there was a significantly greater decrease in parents who rarely/never used soap during handwashing in the intervention villages than in the control district [ROR = 0.49 (0.27–0.92)].

#### 4.7.4 Effect of the intervention on household ownership and quality of WASH-related assets

As Table 7 shows, the odds of owning a utensils rack in the household was found to increase significantly more in the intervention district than in the control [ROR = 3.27 (1.81–5.88)]. Furthermore, there was a significantly greater presence of handwashing facilities outside household latrines in the intervention villages at follow-up; this increase was significant overall when compared to the change in control villages [ROR = 9.66 (1.55–60.15)].

There was an increased number of covered household latrine drop holes in the intervention villages at follow-up; when compared to the control district, the intervention effect was significant [ROR = 2.93 (1.27–6.88)]. Intervention district households were significantly more likely to have a latrine classified as good standard in terms of privacy status (latrine has a standing wall, lockable door, and roof); the intervention effect was generally significant when compared with results from the control district [ROR = 3.73 (1.64–8.53)].

## 5 Discussion and conclusions

Our study contributes to existing literature on community participation in sustaining health outcomes by demonstrating the relevance of village governance structures for integrated NTD-WASH intervention programs in Tanzania. As opposed to earlier initiatives in the country aimed at improving the governance of NTD control program, the EDG model places the village at the centre of the reform process emphasising how efforts to strengthen health program sustainability through community participation invoke issues related to the balance of power and control, for example between program implementers, the SSC and the community as well as between other village-level actors.

Although a pilot project, the enactment of social processes over several months has provided an impetus for resurrecting Tanzania's village health governance structure and shows how health program sustainability may be achieved in terms of finance and processes by catalysing community participation in decision-making and program implementation. We measured public health benefits associated with the intervention and linked these to specific activities which included managing committee functioning, promoting health awareness, self-organisation and data handling, income-generation and interaction between village bodies. Our study supports the use of a mixed methodology approach as a new evaluation paradigm that sheds light on the inner workings of community engagement and its relevance for NTD control. Overall, our findings suggest that the EDG model has resulted in a statistically significant reduction in the prevalence of schistosomiasis diarrhoea although as this was an exploratory study, we recognise its methodological limitations, in particular the quasi-experimental study design adopted; a fully community-randomized controlled trial would allow for statistically more robust conclusions and eliminate the threats to validity inherent in non-randomized studies. It was difficult to randomize intervention to only certain villages in the same ward/district. As the pilot included an educational component (training of SSC and sensitization campaigns), it was possible that people in the same ward/district might apply the knowledge gained to non-intervention villages, thereby potentially masking the true effect of intervention. Nevertheless, the use of a comparison group allowed us to assess the two districts and their similar background factors makes it more likely that our design enables the causal interpretations of observed associations. Other limitations of our study design concerned the fact that exposure to a test could have affected scores on subsequent exposure to that test and the fact that the impact of the intervention was influenced by other events. Although at different levels and stages, as WASH and NTD interventions were ongoing in both districts at the time the project was being implemented, we acknowledge that the decrease of diarrhoea in the control villages could also be explained by the National Sanitation and Hygiene Campaign that was concurrent at the time of our study. However, despite this, we still found many significantly better improvements in the intervention district suggesting the value of the EDG intervention.

The SSCs are part of a shifting landscape in which longer term changes in the way local communities are involved in processes of health governance may be taking root (George et al., 2015b). Nevertheless, our findings raise several critical issues that need to be addressed in the context of the EDG village governance structure which implicate sustainable control of NTDs. First, the imposition of sanitation fines by SSCs was found to be a contentious issue in our intervention villages and this has been corroborated by a study in Eastern Zambia where sanitation fines were rarely followed and resulted in tension between the village headman and community members (Bardosh, 2015). Second, the lack of transparency and accountability within SSCs both in terms of income and expenditure and due to possible corrupt practices related to usage of funds threatens to disrupt the gains from the EDG intervention. Third, to be effective in terms of sustainable village health governance, the SSC needs to develop capacity to monitor health status and income-generation within the village. Fourth, sustainability of the EDG model is reliant on the continued nurturing of horizontal linkages between other village entities such as the village assembly, the village council and the VICOBA groups for outreach to the wider community. Finally, the success of the EDG model remains highly dependent on the perceptions and practices of VHWs who so far have contributed their services on a voluntary basis (Mubyazi and Hutton, 2012). More recently, there is a growing movement to implement sustainable community health worker programs in developing countries, with Tanzania having hosted the global ‘One Million Community Health Worker’ campaign in 2013.^6^

Finally, policy recommendations follow from our study as creating and sustaining community participation implies an important role for the state. There is need for continued support by the government for strengthening village health governance both in terms of capacity-building and in terms of establishing effective mechanisms to integrate the local intelligence that derives from village committees into the planning process (Mansuri and Rao, 2013). Moreover, as the SSCs gain strength, the state needs to ensure that these committees adequately represent all sections of the village population, particularly marginalised groups whilst recognising that these committees may serve to challenge local socio-political structures. Ultimately, as demonstrated in recent studies (Madon and Krishna, 2017), the promotion of community participation for sustainable health outcome requires considerable patience and determination as tactics are tried and new alliances are formed at grassroots level. While this paper was based on a study in Tanzania, we believe that the experience gained from implementing the EDG model could be transferrable to other districts with similar health, social-economic and cultural backgrounds in resource-poor settings.

## Footnotes

1 From 2016 this Ministry is called Ministry of Health, Community Development, Gender, Elderly and Children (MoHCDGEC).

2 The SSC was established in 1982 as a village-level body re-elected every 5 years responsible for health, environment, water and community development including overseeing the functioning of NTD and WASH programs. In Tanzania, VICOBA started in 2009 and operates through self-selected groups of 25–30 members who organise themselves, undergo training in business management and entrepreneurship and are linked to a commercial bank for finance.

3 The Declaration emerged from the World Health Organization international conference on primary healthcare at Alma Ata in 1978.

4 The National Sanitation and Hygiene Campaign was conducted during the cholera outbreak and coincided with our project intervention period.

5 We drew on WASH and the Neglected Tropical Diseases: A global manual for WASH implementers, 2014, www.washntds.org.

6 http://1millionhealthworkers.org/2013/04/15/tanzania-hosts-global-one-million-community-health-workers-campaign-forum/.

## References

[bib1] Amaziqo U.V., Brieger W.R., Katabarwa M., Akogun O., Ntep M., Boatin B., N'Doyo J., Noma M., Seketeli A. (2002). The challenges of community-directed treatment within the african programme for onchocerciasis control (APOC). Ann. Trop. Med. Parasitol..

[bib2] APPMG (2015). UK Coalition against NTDs: Annual Report 2014-15.

[bib3] Banek K., Nankabirwa J., Maiteki-Sebuguzi C.M., DiLiberto D., Taaka L., Chandler C., Staedke S. (2015). Community Case Management of Malaria: exploring support, capacity and motivation of community medicine distributors in Uganda. Health Pol. Plann..

[bib4] Bardosh K. (2014). Global Aspirations, Local Realities: the role of social science research in controlling neglected tropical diseases. Infectious Diseases of Poverty.

[bib5] Bardosh K. (2015). Achieving ‘total sanitation’ in rural african geographies: poverty, participation and pit latrines in eastern Zambia. Geoforum.

[bib6] Campbell C. (2013). Assessing participation in a community-based health planning and services program in Ghana. BMC Health Serv. Res..

[bib7] Dambisya Y.M. (2007). A Review of Non-financial Incentives for Health Worker Retention in East and Southern Africa: Health Systems Research Group.

[bib8] Draper A.K., Hewitt G., Rifkin S. (2010). Chasing the Dragon: developing indicators for the assessment of community participation in health programs. Soc. Sci. Med..

[bib9] Frumence G., Nyamhanga T., Mwangu M., Hurtig A. (2014). Participation in health planning in a decentralised health system: experiences from facility governing committees in the Kongwa district of Tanzania. Global Publ. Health.

[bib10] George A., Mehra V., Scott K., Sriram V. (2015). Community Participation in Health Systems Research: a systematic review assessing the state of research, the nature of interventions involved and the features of engagement with communities. PLoS One.

[bib11] George A., Scott K., Garimella S., Mondal S., Ved R., Sheikh K. (2015). Anchoring Contextual Analysis in Health Policy and Systems Research: a narrative of contextual factors influencing health committees in low and middle income countries. Soc. Sci. Med..

[bib12] Hojsgaard S., Halekoh U., Yan J. (2006). The R package geepak for generalized estimating equations. J. Stat. Software.

[bib13] Hossain S.M., Bhuiya A., Khan A.R., Uhaa I. (2004). Community development and its impact on health: South asian experience. Br. Med. J..

[bib14] Kabataraine N., Malecela M., Lado M., Zaramba S., Amiel O., Kolazinski J.H. (2010). How to or not to integrate vertical program for the control of major neglected tropical diseases in sub-saharan africa. PLoS Neglected Trop. Dis..

[bib15] Lengeler C., Utzinger J., Tanner M. (2002). Questionnaires for rapid screening of schistosomiasis in sub-saharan africa. Bull. World Health Organ..

[bib16] Liang K.Y., Zeger S.L. (1986). Longitudinal data analysis using generalized linear models. Biometrika.

[bib17] Madon S., Krishna S. (2017). Challenges of accountability in resource-poor contexts: lessons about invited spaces from Karnataka's village health committees. Oxf. Dev. Stud..

[bib18] Madon S., Amaguru J., Malecela M., Michael E. (2014). Can mobile phones help control neglected tropical diseases: experiences from Tanzania. Soc. Sci. Med..

[bib19] Mansuri G., Rao V. (2013). Can Participation be Induced? Some evidence from developing countries. Crit. Rev. Int. Soc. Polit. Philos..

[bib20] Mashoto K.O., Malebo H.M., Msisiri E., Peter E. (2014). Prevalence, one week incidence and knowledge on causes of diarrhoea: household survey of under-fives and adults in Mkuranga district, Tanzania. BMC Publ. Health.

[bib21] Malecela M.N., Mwingira U., Mwakitalu M.E., Kabali C., Michael E., Mackenzie C.D. (2013). The Sharp End – experiences from the Tanzanian program for the elimination of lymphatic filariasis: notes from the end of the road. Ann. Trop. Med. Parasitol..

[bib22] Maluka S.O., Bukagile G. (2016). Community Participation in the Decentralised District Health System in Tanzania: why do some health committees perform better than others?. Int. J. Health Plann. Mgmt..

[bib23] Marchal B., Van Dormael M., Pirard M., Cavalli A., Kegels G., Polman K. (2011). Neglected tropical disease (NTD) control in health systems: the interface between programs and general health services. Acta Trop..

[bib24] Mubyazi G.M., Hutton G. (2012). Rhetoric and reality of community participation in health planning, resource allocation and service delivery: a review of reviews, primary publications and grey literature. Rwanda J. Health Sciences.

[bib40] Mubyazi G., Mushi A.K., Shayo E., Mdira K., Ikingura J., Mutagwaba D., Malecela M., Njunwa K.J. (2007). Local primary health care committees and community-based health workers in Mkuranga District, Tanzania: does the public recognise and appreciate them?. Ethno-Med..

[bib25] Mwingira J., Means A.R., Chikawe M., Kilembe B., Lyimo D., Crowley K., Rusibamayila N., Nshala A., Mphuru A. (2016). Integrating neglected tropical disease and immunization programs: the experience of the tanzanian ministry of health. Am. J. Trop. Med. Hyg..

[bib26] Ozawa S., Pongpirul K. (2013). 10 best resources on … mixed methods in health systems. Health Pol. Plann..

[bib27] Pluye P., Potvin L., Denis J.L. (2004). Making public health programs last: conceptualizing sustainability. Eval. Progr. Plann..

[bib28] R Core Team (2014). R: a Language and Environment for Statistical Computing. http://www.R-project.org/.

[bib29] Rifkin S.B. (1986). Lessons from community participation in health programs. Health Pol. Plann..

[bib30] Rifkin S.B., Muller F., Bichmann W. (1988). Primary health care: on measuring participation. Soc. Sci. Med..

[bib31] Rifkin S.B. (2003). A framework linking community empowerment and health equity: it is a matter of CHOICE. J. Health Popul. Nutr..

[bib32] Rifkin S.B. (2014). Examining the links between community participation and health outcomes: a review of the literature. Health Pol. Plann..

[bib33] SCI/MoHSW (2010). Schistosomiasis Baseline Survey Report.

[bib34] Scheirer M., Dearing J. (2011). An agenda for research on the sustainability of public health programs. Am. J. Publ. Health.

[bib35] Shediac-Rizkallah M., Bone L. (1998). Planning for the Sustainability of Community-Based Health Programs: conceptual frameworks and future directions for research, practice and policy. Health Educ. Res..

[bib36] Shigayeva A., Coker R. (2015). Communicable disease control program and health systems: an analytical approach to sustainability. Health Pol. Plann..

[bib37] Singh P. (2013). Community Health Workers: a local solution to a global problem. N. Engl. J. Med..

[bib38] Toledo Romani M., Vanlerberghe V., Perez D., Lefevre P., Ceballor E., Bandera D., Baly Gil A., Van der Stuyft P. (2007). Achieving Sustainability of Community-based Dengue Control in Santiago de Cuba. Soc. Sci. Med..

[bib39] World Health Organization (2002). Community Participation in Local Health and Sustainable Development: Approaches and Techniques.

