# Supplementary material for: The role of community participation for sustainable integrated neglected tropical diseases and water, sanitation and hygiene intervention programs: A pilot project in Tanzania
Source: Soc Sci Med. 2018 Apr;202:28–37. doi: 10.1016/j.socscimed.2018.02.016 (PMC5906643; doi:10.1016/j.socscimed.2018.02.016)
Supplement: Supplementary material [file mmc1.docx]

**Appendix**

A: Methodology for the classification of schistosomiasis

In this study, we sought to classify individuals as schistosomiasis positive or negative using a questionnaire. We conducted a meta-analysis to determine the sensitivity and specificity of questions suggested in a review by Lengeler et al. (Lengeler, Utzinger, & Tanner, 2002) to predict the schistosomiasis status of an individual.

While the meta-analysis produced the sensitivity and specificity of individual questions for correctly classifying schistosomiasis infection (as summarized in Tables S1 and S2), given that multiple individual questions were asked for each disease, we also sought to determine the sensitivity and specificity of answering positively to combinations of questions. The sensitivity and specificity for combined questions were obtained by evaluating the test questions in series, meaning an individual would have to answer positively to each question to be deemed positive for that group of test questions. Sensitivities for questions asked in series were multiplied together: ${{se}_{q1 and q2}= se}_{q1}* {se}_{q2}.$ Specificities for questions asked in series were computed using the following formula: ${{sp}_{q1 and q2}= 1-(1-sp}_{q1})*(1-{sp}_{q2}).$

After we computed the sensitivity and specificity values for individual questions from the meta-analysis and the sensitivity and specificity values for combinations of questions utilizing the equations for questions asked in series, we computed one final value for each question and combination of questions that combined the sensitivity and specificity values: the positive likelihood ratio (LR+). We calculated the LR+ for each question and combination of questions using the formula$LR+ = \frac{sensitivity}{1-specificity}$. The LR+ provides an estimate of how many times more likely individuals with the target disease are to test positive than individuals without the target disease (Deeks & Altman, 2004). For this paper, the criteria of LR+>3.5 was used as the cut-off, with tests exceeding a LR+ of 3.5 being considered in classifying infections status. Individuals positive for at least one of the tests with a LR+ >3.5 were classified as ‘infected’ for the purposes of analysis. Individuals found negative for all the tests with a LR+ >3.5 were classified as ‘uninfected’. In the literature, LR+ values > 2 have been used for indicating disease status, with prognosis of disease generally increasing with LR+ values (Jaeschke, Guyatt, & Sackett, 1994).

**Table S1:** Diagnostic performance of each question and combination of questions for correctly classifying an individual as *S. haematobium-*positive

| **S. haematobium diagnostic question(s)** | **Sensitivity^1^** | **Specificity^1^** | **LR+^2^** |
| --- | --- | --- | --- |
| Blood in urine | 0.508 | 0.868 | 3.85**^3^** |
| Pain during urination | 0.445 | 0.818 | 2.45 |
| History of schistosomiasis infection | 0.365 | 0.807 | 1.89 |
| Blood in urine  Pain during urination | 0.226 | 0.976 | 9.41**^3^** |
| Blood in urine  History of schistosomiasis infection | 0.185 | 0.975 | 7.28**^3^** |
| Pain during urination  History of schistosomiasis infection | 0.162 | 0.965 | 4.62**^3^** |
| Blood in urine  Pain during urination  History of schistosomiasis infection | 0.083 | 0.995 | 17.80**^3^** |

^1^ Sensitivity and specificity for individual questions computed from meta-analysis; sensitivity and

specificity for combination of questions combined in series

^2^ LR+: Positive likelihood ratio

^3^ Indicates LR+ >3.5 and subsequent positive schistosomiasis classification for this study

**Table S2:** Diagnostic performance of each question and combination of questions for correctly classifying an individual as *S. mansoni*-positive

| **S. mansoni diagnostic question(s)** | **Sensitivity^1^** | **Specificity^1^** | **LR+^2^** |
| --- | --- | --- | --- |
| Blood in stool | 0.249 | 0.904 | 2.69 |
| Bloody diarrhea | 0.283 | 0.849 | 1.87 |
| Abdominal pain | 0.399 | 0.666 | 1.19 |
| History of schistosomiasis | 0.329 | 0.765 | 1.40 |
| Blood in stool  Bloody diarrhea | 0.070 | 0.986 | 4.86**^3^** |
| Blood in stool  Abdominal pain | 0.099 | 0.968 | 3.10 |
| Blood in stool  History of schistosomiasis | 0.082 | 0.977 | 3.63**^3^** |
| Bloody diarrhea  Abdominal pain | 0.113 | 0.950 | 2.24 |
| Bloody diarrhea  History of schistosomiasis | 0.093 | 0.965 | 2.62 |
| Abdominal pain  History of schistosomiasis | 0.131 | 0.922 | 1.67 |
| Blood in stool  Bloody diarrhea  History of schistosomiasis | 0.023 | 0.997 | 6.81**^3^** |
| Blood in stool  Bloody diarrhea  Abdominal pain | 0.028 | 0.995 | 5.81**^3^** |
| Blood in stool  History of schistosomiasis  Abdominal pain | 0.033 | 0.992 | 4.34**^3^** |
| Bloody diarrhea  History of schistosomiasis  Abdominal pain | 0.037 | 0.988 | 3.13 |
| Blood in stool  Bloody diarrhea  History of schistosomiasis  Abdominal pain | 0.009 | 0.999 | 8.13**^3^** |

References

Deeks, J. J., & Altman, D. G. (2004). Diagnostic tests 4: likelihood ratios. *BMJ (Clinical Research Ed.)*, *329*(7458), 168–9. https://doi.org/10.1136/bmj.329.7458.168

Jaeschke, R., Guyatt, G. H., & Sackett, D. L. (1994). Users’ guides to the medical literature. III. How to use an article about a diagnostic test. B. What are the results and will they help me in caring for my patients? The Evidence-Based Medicine Working Group. *JAMA*, *271*(9), 703–7.

Lengeler, C., Utzinger, J., & Tanner, M. (2002). Questionnaires for rapid screening of schistosomiasis in sub-Saharan Africa. *Bulletin of the World Health Organization*, *80*(3), 235–242.

B: Details of the GEE model employed

The GEE model employed may be described as follows (Liang & Zeger, 1986):

g(μ_i_)=X_i_^T^ β

where g is a known link function, β is an unknown p x 1 vector of regression coefficients, and X_i_ is a p x 1 vector of covariates (Wang, 2014). The specific model we used can be described as:

$$\mathrm{logit}\left( \mu\right)=c+ \beta_{1}G+ \beta_{2}T+\beta_{3}(GT)$$

Where $c$ is the the intercept, $G$ is the treatment group (1= intervention; 0 = control); and $T$ is the time (1= follow-up; 0 = baseline).

The variance in this model has a random component related to the type of data being modelled (binomial in the present case), and a co-variance structure that is used to incorporate two intraclass correlations in the data: cross-sectional within cluster correlation as well as correlation in outcomes over time (Preisser et al., 2003).

References

Liang, K. Y., & Zeger, S. L. (1986). Longitudinal data analysis using generalized linear models. Biometrika, 73(1), 13-22.

Wang, M. (2014). Generalized Estimating Equations in Longitudinal Data Analysis: A Review and Recent Developments. Advances in Statistics, 2014.

Preisser, J. S., Young, M. L., Zaccaro, D. J., & Wolfson, M. (2003). An integrated population‐averaged approach to the design, analysis and sample size determination of cluster‐unit trials. Statistics in medicine, 22(8), 1235-1254.

C: Baseline Factors of Control and Intervention districts

Due to baseline factors such as sex, age, and socio-economic status (see below), being found to not significantly differ between the control and intervention districts, we did not adjust for confounders in the model and have added a sentence providing this explanation to the quantitative methods section of the paper.

| Characteristic | Rufiji  n (%) | Mkuranga  n(%) | Chi-squared statistic | p-value |
| --- | --- | --- | --- | --- |
| Total Participants |  |  |  |  |
| Sex |  |  | 0.03 | 0.90 |
| Male | 361 (40.4) | 324 (40.0) |  |  |
| Female | 533 (59.6) | 486 (60.0) |  |  |
| Age |  |  | 0.9 | 0.30 |
| <13 years old | 399 (44.6) | 380 (46.9) |  |  |
| ≥ 13 years old | 495 (55.4) | 430 (53.1) |  |  |
|  |  |  | Wilcox test-statistic | p-value |
| Socio-economic score* |  |  | 4e05 | 0.700 |
|  | -0.0739 | 0.0816 |  |  |

# *The socio-economic score was calculated using principal components analysis on ownership of 9 binary asset variables (house, land, latrine, motorcycle, bicycle, phone, fridge, TV, and radio). Due to skewed distributions, we used the Mann-Whitney Wilcoxon test to test for differences in mean socio-economic score between the two districts.

D: Specific questions used for comparing knowledge of SSC members and the community on NTD-WASH before and after the EDG model intervention

**QUESTIONNAIRE**

**Name ________________________________ Household ID number__________________**

**Village__________________________________Ward________________________**

**Sex___________________________________Age in years_______________**

1. **For each sentence put tick (√) in Yes or No box if you are agree or disagree with the sentence**

| *I. Water contamination* | **Yes** | **No** |
| --- | --- | --- |
| 1. Throwing dirty in water source makes water unsafe |  |  |
| 1. Bathing in water bodies contaminate water sources |  |  |
| 1. Bathing near water sources lead to contamination |  |  |
| 1. Handling water with dirty hands lead to contamination |  |  |
| 1. Uncovered water is not safe for drinking |  |  |
| 1. Water sources are contaminated through open defecation |  |  |
| 1. It is important to use latrines for prevention of diseases caused by soil and water contamination |  |  |
| *II. Sanitation knowledge for disease prevention* |  |  |
| 1. Washing of soiled clothing/bedding and face prevent trachoma |  |  |
| 1. Hand washing prevent STHs |  |  |
| 1. Wearing shoes outside prevent STHs |  |  |
| 1. Use of unsafe water for bathing, clothes washing, and swimming cause schistosomiasis |  |  |
| 1. Avoiding physical contact with contaminated surface water prevent schistosomiasis |  |  |

1. **The following are common NTDs in Rufiji district. For each disease put tick (√) in Yes or No box if you are agree or disagree with the sentence respectively**

| *Knowledge on common NTDs occurring in Rufiji* | **Yes** | **No** |
| --- | --- | --- |
| 1. Malaria |  |  |
| 1. Hydrocele |  |  |
| 1. Elephantiasis |  |  |
| 1. STHs |  |  |
| 1. Trachoma |  |  |
| 1. Schistosomiasis |  |  |
| 1. Leprosy |  |  |
| 1. Cholera |  |  |

1. **Consumption of unsafe water cause the following diseases. For each disease put tick (√) in Yes or No box if you are agree or disagree with the sentence respectively**

| *Hygiene for disease prevention* | **Yes** | **No** |
| --- | --- | --- |
| 1. Malaria |  |  |
| 1. Typhoid |  |  |
| 1. Diarrhea |  |  |
| 1. Elephantiasis |  |  |
| 1. Cholera |  |  |
| 1. Trachoma |  |  |
| 1. Schistosomiasis |  |  |

1. **Use of unsafe water for bathing, clothes washing and swimming cause the following diseases. For each disease put tick (√) in Yes or No box if you are agree or disagree**

| *Sanitation for disease prevention* | **Yes** | **No** |
| --- | --- | --- |
| 1. Malaria |  |  |
| 1. Typhoid |  |  |
| 1. Diarrhea |  |  |
| 1. Elephantiasis |  |  |
| 1. Cholera |  |  |
| 1. Trachoma |  |  |
| 1. Schistosomiasis |  |  |

1. **Mosquito cause the following diseases. For each disease put tick (√) in Yes or No box if you are agree or disagree**

| *Disease transmission* | **Yes** | **No** |
| --- | --- | --- |
| 1. Malaria |  |  |
| 1. Typhoid |  |  |
| 1. Diarrhea |  |  |
| 1. Elephantiasis |  |  |
| 1. Cholera |  |  |
| 1. Trachoma |  |  |
| 1. Schistosomiasis |  |  |

1. **Flies cause the following diseases. For each disease put tick (√) in Yes or No box if you are agree or disagree**

| *Disease transmission* | **Yes** | **No** |
| --- | --- | --- |
| 1. Malaria |  |  |
| 1. Typhoid |  |  |
| 1. Diarrhea |  |  |
| 1. Elephantiasis |  |  |
| 1. Cholera |  |  |
| 1. Trachoma |  |  |
| 1. Schistosomiasis |  |  |

1. **The following are neglected tropical diseases which may lead to disability. For each disease put tick (√) in Yes or No box if you are agree or disagree**

| *Diseases causing disability* | Yes | No |
| --- | --- | --- |
| 1. Malaria |  |  |
| 1. Typhoid |  |  |
| 1. Diarrhea |  |  |
| 1. Elephantiasis |  |  |
| 1. Cholera |  |  |
| 1. Trachoma |  |  |
| 1. Schistosomiasis |  |  |

1. **Drinking safe water prevent the following diseases. For each disease put tick (√) in Yes or No box if you are agree or disagree**

| *Hygiene for diseases prevention* | **Yes** | **No** |
| --- | --- | --- |
| 1. Malaria |  |  |
| 1. Typhoid |  |  |
| 1. Diarrhea |  |  |
| 1. Elephantiasis |  |  |
| 1. Cholera |  |  |
| 1. Trachoma |  |  |
| 1. Schistosomiasis |  |  |

1. **Washing hands have prevention impact on the following diseases. For each disease put tick (√) in Yes or No box if you are agree or disagree**

| *Hygiene for diseases prevention* | **Yes** | **No** |
| --- | --- | --- |
| 1. Malaria |  |  |
| 1. Typhoid |  |  |
| 1. Diarrhea |  |  |
| 1. Elephantiasis |  |  |
| 1. Cholera |  |  |
| 1. Trachoma |  |  |
| 1. Schistosomiasis |  |  |

1. **Increasing access to sufficient amounts of safe water for personal hygienic purposes (e.g., washing hands, face, or body, bathing, and doing laundry). For each disease put tick (√) in Yes or No box if you are agree or disagree**

| *Water intervention for disease prevention* | **Yes** | **No** |
| --- | --- | --- |
| 1. Malaria |  |  |
| 1. Typhoid |  |  |
| 1. Diarrhea |  |  |
| 1. Elephantiasis |  |  |
| 1. Cholera |  |  |
| 1. Trachoma |  |  |
| 1. Schistosomiasis |  |  |

1. **Increasing access to sufficient amounts of safe water for environmental sanitation (e.g., cleaning latrines). For each disease put tick (√) in Yes or No box if you are agree or disagree**

| *Water intervention for disease prevention* | **Yes** | **No** |
| --- | --- | --- |
| 1. Malaria |  |  |
| 1. Typhoid |  |  |
| 1. Diarrhea |  |  |
| 1. Elephantiasis |  |  |
| 1. Cholera |  |  |
| 1. Trachoma |  |  |
| 1. Schistosomiasis |  |  |

1. **Increasing access to safe water for drinking/food preparation. For each disease put tick (√) in Yes or No box if you are agree or disagree**

| *Water intervention for disease prevention* | **Yes** | **No** |
| --- | --- | --- |
| 1. Malaria |  |  |
| 1. Typhoid |  |  |
| 1. Diarrhea |  |  |
| 1. Elephantiasis |  |  |
| 1. Cholera |  |  |
| 1. Trachoma |  |  |
| 1. Schistosomiasis |  |  |

1. **Monitoring impact of water resource development, waste water management, and sanitation programs on vector breeding levels prevent the following diseases. For each disease put tick (√) in Yes or No box if you are agree or disagree**

| *Water intervention for disease prevention* | **Yes** | **No** |
| --- | --- | --- |
| 1. Malaria |  |  |
| 1. Hydrocele |  |  |
| 1. Elephantiasis |  |  |
| 1. STHs |  |  |
| 1. Trachoma |  |  |
| 1. Schistosomiasis |  |  |

1. **Reducing open defecation prevent the following diseases. For each disease put tick (√) in Yes or No box if you are agree or disagree**

| *Sanitation for disease prevention* | **Yes** | **No** |
| --- | --- | --- |
| 1. Malaria |  |  |
| 1. Hydrocele |  |  |
| 1. Elephantiasis |  |  |
| 1. STHs |  |  |
| 1. Trachoma |  |  |
| 1. Schistosomiasis |  |  |

1. **How availability and use of safe water is important in prevention of NTDs**
2. Not important at all
3. Not important
4. Little importance
5. Very important
6. **How important is sanitation in prevention of NTDs**
7. Not important at all
8. Not important
9. Little important
10. Very important
11. **How important is hygiene in prevention of NTDs**
12. Not important at all
13. Not important
14. Little important
15. Very important
